# Ablation of Premature Ventricular Complexes Triggering Ventricular Fibrillation in a Patient with Long QT Syndrome

**Published:** 2011-05-01

**Authors:** Juan Jose Sanchez-Munoz, Arcadi Garcuia-Alberola, Juan Martinez-Sanchez, Esperanza Garcia-Molina, Mariano Valdes-Chavarri

**Affiliations:** 1 Arrhythmia Unit, Service of Cardiology, University Hospital Virgen de la Arrixaca de Murcia, Spain; 2 Molecular Biology Research Unit, University Hospital Virgen de la Arrixaca. Murcia, Spain

**Keywords:** Ablation, ventricular fibrillation

## Abstract

We describe the case of a patient with long QT syndrome   and  recurrent  ventricular fibrillation, triggered by premature ventricular complexes (PVCs) with a left bundle branch block pattern and inferior axis of the QRS. Activation mapping demonstrated the origin of the PVCs to be in the right ventricular outflow tract. Ventricular fibrillation (VF) was successfully treated by catheter ablation of the triggering PVCs and there has been no recurrence of VF during a follow-up period of 14 months.

## Case

**Figure 1:**
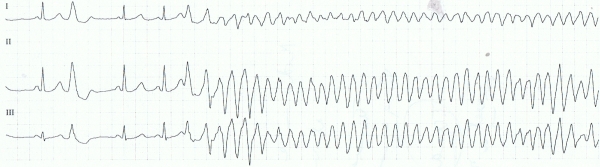
Ventricular fibrillation triggered by premature ventricular complex

A 56 year old female was admitted in hospital, with episodes of agonal nocturnal respiration, showing polymorphic ventricular tachycardia which degenerated into ventricular fibrillation (VF). There were no palpitations or syncope in her past medical history and no family history of sudden death. Recurrent episodes of VF (Figure 1), always in the night, appeared over the following days, requiring repeated external defibrillation.

**Figure 2:**
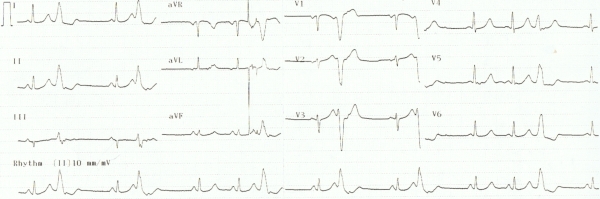
Twelve-lead ECG with RVOT premature beat and long QT interval

A 12-lead ECG revealed sinus rhythm and frequent premature ventricular complexes (PVCs) with a left bundle branch block (LBBB) pattern and inferior axis of the QRS. The mean coupling interval was 360 ± 30 ms. QTc interval was 489 ms (Figure 2). All the episodes of VF were triggered by PVCs. Echocardiography revealed normal right and left ventricular ejection fraction and  a normal coronary angiography excluded myocardial ischemia. Pharmacologic provocation did not show evidence of Brugada syndrome. LQTS was diagnosed based on published diagnostic criteria [1], with a score of five (QT  470 ms, spontaneous VF, syncope), considered as high probability of LQTS. Genetic testing, performed afterwards, did not identify diagnostic channel mutations associated with LQTS, (KCNQ1, KCNH2, SCN5A, KCNE1, KCNE2, KCNJ2), but showed three common  polymorphisms (E1061E, D1819D at the SCN5A gene, and S38G at the KCNE1).

The episodes of VF could not be suppressed by different drugs such as beta-blockers, amiodarone at another centre and the patient was referred to us for an electrophysiology study and catheter ablation.

### Mapping and catheter ablation procedures

**Figure 3:**
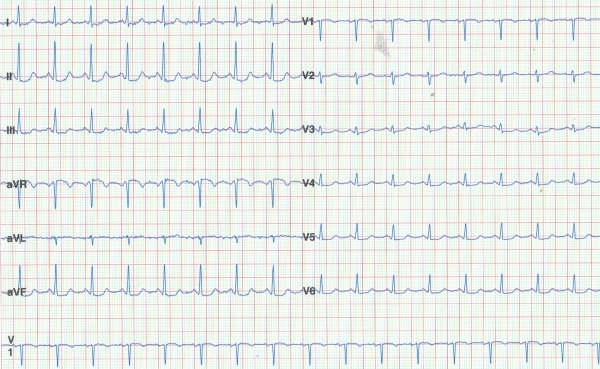
ECG during follow up without PVC

A quadripolar catheter was inserted via the femoral vein and positioned in the right ventricle. A 7-French deflectable ablation catheter with a 4 mm tip electrode (Cordis Webster) was used for mapping and radiofrequency current application. Frequent PVCs with an LBBB morphology and inferior axis were present during the study and allowed us to perform activation sequence mapping using CARTO mapping system demonstrating the origin of the PVCs to be in the posterior septal wall of right ventricular outflow tract (RVOT). PVCs were not associated with the Purkinje system. Three applications of radiofrequency (65º C, 50 W, 60 s) were performed at the site with the earliest activation and led to complete cessation of PVCs. A defibrillator was implanted in view of persistent long QTc interval and risk of sudden death despite ablation of triggering PVCs. There has been no recurrence of VF during a follow-up period of 14 months, and QTc was 472 ms (Figure 3).

## Discussion

There are a few reports of patients with LQTS having undergone ablation for VF by eliminating premature beats [2,3]. Only one of them showed PVCs originating from RVOT, as it did in our case in the rest Purkinje activity preceded the PVC. Though RVOT PVCs triggering VF is somewhat logical in patients with Brugada Syndrome where a specific arrhythmogenic area in the right ventricle has been suggested [4], its occurrence  in idiopathic VF or LQTS is interesting. Ventricular fibrillation can be successfully treated by catheter ablation of the PVC trigger in patients with LQTS.
